# Theoretical Prediction and Experimental Synthesis of Zr_3_AC_2_ (A = Cd, Sb) Phases

**DOI:** 10.3390/ma17071556

**Published:** 2024-03-28

**Authors:** Jia Luo, Fengjuan Zhang, Bo Wen, Qiqiang Zhang, Longsheng Chu, Yanchun Zhou, Qingguo Feng, Chunfeng Hu

**Affiliations:** 1 Key Laboratory of Advanced Technologies of Materials, Ministry of Education, School of Materials Science and Engineering, Southwest Jiaotong University, Chengdu 610031, China; luojia07232000@163.com (J.L.); zfj6782022@163.com (F.Z.); swjtu202213@163.com (B.W.); zhangqiqiang@std.uestc.edu.cn (Q.Z.); lshchu@home.swjtu.edu.cn (L.C.); 2 School of Materials Science and Engineering, Zhengzhou University, Zhengzhou 450001, China

**Keywords:** Zr_3_CdC_2_, Zr_3_SbC_2_, first-principles calculation, theoretical mechanical properties, synthesis

## Abstract

MAX phases have great research value and application prospects, but it is challenging to synthesize the MAX phases containing Cd and Sb for the time being. In this paper, we confirmed the existence of the 312 MAX phases of Zr_3_CdC_2_ and Zr_3_SbC_2_, both from theoretical calculations and experimental synthesis. The Zr_3_AC_2_ (A = Cd, Sb) phase was predicted by the first-principles calculations, and the two MAX phases were confirmed to meet the requests of thermal, thermodynamic, and mechanical stabilities using formation energy, phonon dispersion, and the Born–Huang criteria. Their theoretical mechanical properties were also systematically investigated. It was found that the elastic moduli of Zr_3_CdC_2_ and Zr_3_SbC_2_ were 162.8 GPa and 164.3 GPa, respectively. Then, differences in the mechanical properties of Zr_3_AC_2_ (A = Cd, In, Sn, and Sb) were explained using bond layouts and charge transfers. The low theoretical Vickers hardness of the Zr_3_CdC_2_ (5.4 GPa) and Zr_3_SbC_2_ (4.3 GPa) phases exhibited excellent machinability. Subsequently, through spark plasma sintering, composites containing Zr_3_CdC_2_ and Zr_3_SbC_2_ phases were successfully synthesized at the temperatures of 850 °C and 1300 °C, respectively. The optimal molar ratio of Zr:Cd/Sb:C was determined as 3:1.5:1.5. SEM and the EDS results analysis confirmed the typical layered microstructure of Zr_3_CdC_2_ and Zr_3_SbC_2_ grains.

## 1. Introduction

Nowotny discovered the H-phases in 1967, and Barsoum defined this kind of material as the M*_n_*_+1_AX*_n_* phase (MAX phase) until 2000 [1,2,3,4]. M*_n_*_+1_AX*_n_* phases are a family of hexagonal, ternary layered compounds. Generally, M-site elements are early transition metals, A-site elements mainly come from ⅢA-ⅤA elements, and X-site elements are C, N, or B. All MAX phases have a hexagonal crystal structure, and their space group belongs to *P*6_3_*/mmc*. The X atom is located at the center of the octahedron composed of M atoms, and the entire crystal structure is composed of densely packed M_6_X octahedron layers and A layers, which are alternated periodically. According to the difference in the number of M layers between two A layers, MAX phases can be generally divided into 211 (*n* = 1), 312 (*n* = 2), and 413 (*n* = 3) phases [5,6,7,8,9,10,11,12,13]. The MAX phase contains covalent, ionic, and metallic bonds and has both ceramic and metallic properties [14]. The strong covalent and ionic bonds between MX atoms contribute to the thermodynamic and mechanical stability of the MAX phase [15]. The weak covalent bonds and metal bonds exist between MA atoms, and metal bonds exist between MM atoms. The special nanolayered crystal structure endows them with a high elastic modulus, high strength, and excellent machinability, as well as high electrical conductivity and high thermal conductivity. Therefore, MAX phases are promising to be high-temperature structural materials, electrode brush materials, chemical resistant materials, high-temperature heating materials, etc. [16,17].

Nowadays, MAX phases are becoming popular research materials due to their excellent properties and wide range of potential applications [18]. In recent years, more and more 312 MAX phases have been discovered and studied. For instance, 312 MAX phases containing In and Sn as A-position elements have been successfully synthesized recently. Lapauw et al. successfully synthesized Zr_3_SnC_2_ by spark plasma sintering with Fe, Co, or Ni additives in 2017 and proposed the mechanism for the synthesis of Zr_3_SnC_2_ [19,20]. Most recently, an indium-containing MAX phase of Zr_3_InC_2_ was also successfully sintered by spark plasma sintering [21]. In the periodic table of elements, Cd and Sb are in the same period of In and Sn, and these four elements are located next to each other. However, perhaps due to the challenging synthesis of MAX phases in which the A elements are Cd and Sb, we found that previous research reports on MAX phases containing Cd and Sb were uncommon. For example, Ti_2_CdC was successfully sintered at 750 °C by Nowotny in 1964 [22]. The crystal structure, chemical bonding, and elastic properties of Ti_2_CdC were investigated by first-principles calculations, and this cadmium-containing MAX phase was predicted to have potential applications as electric friction materials [23,24]. Therefore, theoretical prediction and experimental synthesis of Zr_3_AC_2_ (A = Cd, Sb) MAX phases is a necessary and meaningful endeavor. To the best of our knowledge, this article is the first report on Zr_3_CdC_2_ and Zr_3_SbC_2_ phases. The discovery of these two MAX phases may provide ideas for the synthesis of high-purity Zr_3_CdC_2_ and Zr_3_SbC_2_ phases and new options in the field of electrically and thermally conductive materials.

In this work, the new 312 MAX phases of Zr_3_AC_2_ (A = Cd, Sb) were predicted to be stable by using the first-principles calculations, and their basic mechanical properties were systematically calculated. Furthermore, by adopting spark plasma sintering, the Zr_3_CdC_2_ and Zr_3_SbC_2_ phases were successfully synthesized, and their laminar microstructures were well determined.

## 2. Experimental Procedures

### 2.1. First-Principles Calculation

In this work, all the calculations were performed by the density functional theory (DFT) calculations in a framework of generalized gradient approximation with the projector augmented-wave method implemented in the Vienna ab initio simulation package (VASP.5.4.4, Hafner Group, University of Vienna, Vienna, Austria), where the Perdew–Burke–Ernzerhof (PBE) functional has been adopted for exchange-correlation potential [25,26]. The convergence criteria for energy and force were set to 10^−6^ eV and 0.01 eV/Å, respectively. The plane-wave basis set cutoff was 450 eV after the convergent test, and the special k-points sampling integration over the Brillouin zone was employed using the Monkhorst–Pack method with 12 × 2 × 2 spatial k-points mesh. The elastic constants, mechanical moduli, and phonon dispersion were calculated through the finite displacement method. In order to obtain an accurate acoustic vibration mode, we first optimized the lattice structure of the MAX phase. Secondly, the mechanical constants were calculated using density functional perturbation theory (DFPT). Finally, lattice vibration and thermodynamic properties were extracted from the VASP output by the Phonopy post-processing 2.17.1 software. Because MAX phases had a layered structure, the van der Waals force was involved through the Grimme-D3 scheme to correctly address the interactions between layers. This method can more accurately describe the weak interaction between molecules by introducing additional van der Waals terms [27]. Additionally, the crystal structures of the Zr_3_AC_2_ (A = Cd, Sb) phases were visualized by using the VESTA-gtk3 software [28].

### 2.2. Synthesis of Zr_3_CdC_2_ and Zr_3_SbC_2_ Phases

Commercial powders of zirconium (99.5% purity, 500 mesh; Eno High-Tech Material Development Co., Ltd., Qinhuangdao, China), cadmium (99.5% purity, 300 mesh; Eno High-Tech Material Development Co., Ltd., China), antimony (99.9% purity, 500 mesh; Eno High-Tech Material Development Co., Ltd., China), and graphite (99.95%, 500 mesh; Eno High-Tech Material Development Co., Ltd., China) were used as the initial materials. To successfully synthesize the Zr_3_CdC_2_ and Zr_3_SbC_2_ phases, different sintering temperatures and molar ratios were designed. The powders were weighed using an electric balance with an accuracy of 10^−4^ g and mixed in a plastic bottle for 12 h at 50 rpm. Then, the mixture was placed into a graphite die with a diameter of 20 mm. The sintering process was carried out in a spark plasma sintering furnace (SPS-20T-10, Shanghai Chenhua Technology Co., Ltd., Shanghai, China). The heating rate was 50 °C/min, and the applied pressure was 30 MPa. After sintering, the samples were cooled down to ambient temperature with the furnace. The surface contaminations of samples were removed by a diamond grinding wheel.

### 2.3. Composition and Microstructure Characterization

X-ray diffraction (XRD) (Ultima IV, Rigaku, Japan) with a Cu-Kα radiation source in the 2*θ* range of 5–75° was used for the composition analyses of the Zr_3_CdC_2_ and Zr_3_SbC_2_ phases. The theoretically calculated X-ray diffraction patterns of Zr_3_CdC_2_ and Zr_3_SbC_2_ were determined by the Materials Studio 8.0 software (Accelrys Software Inc., San Diego, CA, USA) using the optimized lattice parameters of Zr_3_CdC_2_ and Zr_3_SbC_2_ as the input. The microstructure features and elemental compositions of the fracture surfaces of sintered samples were monitored using a scanning electron microscope (SEM) (Apreo 2C, Thermo Fisher Scientific, Brno, Czech Republic) equipped with an energy dispersive spectroscope (EDS) (Oxford ultim Max 65, Oxford Ins., Oxford, UK).

## 3. Results and Discussion

### 3.1. Stability Calculation of Zr_3_CdC_2_ and Zr_3_SbC_2_ Phases

**Figure 1:**
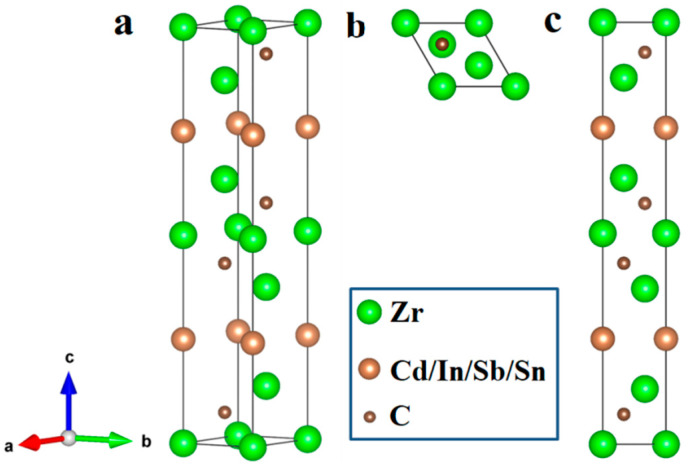
Crystal structures of Zr_3_SbC_2_ and Zr_3_CdC_2_ phases: (**a**) 3-dimensional image, (**b**) top view image ((0001) plane), and (**c**) side view image ((1–100) plane).

The crystal structures of experimentally synthesized Zr_3_SnC_2_ and Zr_3_InC_2_ are both *P*6_3_*/mmc* [19,21]. Since Cd, In, Sn, and Sb belong to the same period in the periodic table of the elements, it was predicted that the properties and crystal structures of Zr_3_CdC_2_ and Zr_3_SbC_2_ are similar to those of Zr_3_SnC_2_ and Zr_3_InC_2_, and therefore, they were modeled and calculated according to the *P*6_3_*/mmc* space group. Figure 1 shows the crystal structures of two new 312 MAX phases of Zr_3_CdC_2_ and Zr_3_SbC_2_. The optimized lattice parameters of Zr_3_AC_2_ (A = Cd, In, Sn, Sb) were calculated and are listed in Table 1. For comparison, the experimental parameters of two synthesized 312 phases of Zr_3_InC_2_ and Zr_3_SnC_2_ are also listed. It is clearly determined that when the M and X sites are identical, the planar lattice parameter *a* shows a monotonic increase from 3.319 to 3.367 Å with the increasing order of the atomic number of A-site elements, while the vertical lattice parameter *c* shows a decrease simultaneously from 20.393 to 19.413 Å, indicating that the bonds within the (0001) plane become weaker and the interlayer interactions become stronger when the A-site elements go from Cd to In to Sn and further to Sb. Additionally, it is known that if the crystals of Zr_3_CdC_2_ and Zr_3_SbC_2_ are stable, they must meet the requests of thermal, thermodynamic, and mechanical stabilities. Therefore, the thermal stability was quantized by the formation energy:

(1) $$\Delta H=\frac{H_{crystal}-lH_{M}-mH_{A}-nH_{X}}{l+m+n}$$

where *M*, *A*, and *X* are composite elements; *l*, *m*, *n* are the number of each element in the unit cell; *H_crystal_* is the enthalpy of the unit cell; and *H_M_*, *H_A_*, and *H_X_* are the corresponding enthalpies of the composite atom in the MAX phases of Zr_3_CdC_2_ and Zr_3_SbC_2_. The calculated formation energies (Δ*H*) of the Zr_3_CdC_2_ and Zr_3_SbC_2_ phases were −1.104 and −1.306 eV/atom, respectively. Therefore, both the formation energies of the Zr_3_CdC_2_ and Zr_3_SbC_2_ phases are negative, corresponding to the thermal stability.

**Figure 2:**
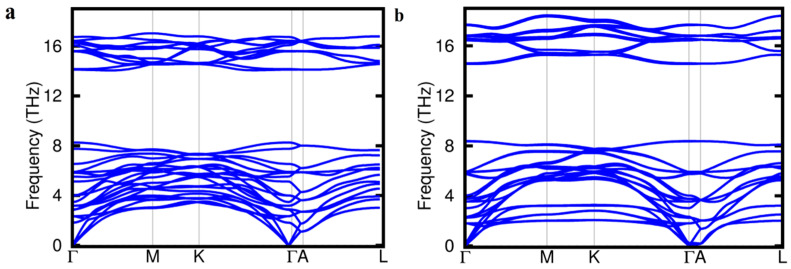
Calculated phonon dispersion of (**a**) Zr_3_SbC_2_ and (**b**) Zr_3_CdC_2_ phases.

Furthermore, the thermodynamic stability of the Zr_3_CdC_2_ and Zr_3_SbC_2_ phases was investigated by calculating the phonon dispersion, as shown in Figure 2. The phonon dispersion curve can reflect the dynamic stability of materials to a certain extent. In the first-principles calculation, high symmetry points were selected in the Brillouin zone according to the crystal structure to calculate the mechanical constants. The calculation results showed that there was no negative frequency (imaginary frequency) in the Brillouin zone, which means that the dynamic stability of materials is studied under standard pressure. The minor softness around the Γ and I points are deemed to be caused by the numerical error in calculations but not by the solid-state physics theory. It is observed that Zr_3_SbC_2_ has a smaller maximum frequency than Zr_3_CdC_2_, which means that Zr_3_CdC_2_ has a higher melting point, i.e., the highest frequency mode is more difficult to activate for Zr_3_CdC_2_ [29]. The calculated melting point of Zr_3_CdC_2_ (1548.344 K) is higher than that of Zr_3_SbC_2_ (1451.880 K). In addition, it is known that the Debye temperature is connected to the lattice vibration, thermal expansion coefficient, specific heat, and melting point of the crystal. As the maximum frequency mode of vibration, the calculated T_D_ using elastic modulus is specified as one of the standard approaches [30,31]. The calculated Debye temperatures of Zr_3_CdC_2_ and Zr_3_SbC_2_ are 392.7 K and 386.2 K, respectively, lower than those of Zr_3_InC_2_ (488.9 K) and Zr_3_SnC_2_ (493.7 K).

On the other hand, the crystals of Zr_3_CdC_2_ and Zr_3_SbC_2_ are also mechanically stable and are confirmed to meet the Born–Huang criteria (Equation (2)), where C*_ij_* is presenting the elastic constants and is listed in Table 2 [32]:

(2) $$C_{11}>|C_{12}|,2C_{13}^{2}<C_{33}(C_{11}+C_{12}),C_{44}>0$$

It is seen that the pure shear elastic constants of C_44_ are lower than the unidirectional elastic constants of C_11_ and C_33_. This result means that the shear deformation is easier to occur in comparison with linear compression along the crystallographic *a*- and *c*-axes. The unequal values of C_11_, C_33_, and C_44_ (C_11_ ≠ C_33_ ≠ C_44_) imply different atomic arrangements and hence, different bonding strengths along the *a*-axis, *c*-axis, and shear planes. The combination of C_12_ and C_13_ leads to functional stress along the crystallographic *a*-axis when a uniaxial strain exists in both the *b*- and *c*-axes. The low values of these constants imply that the Zr_3_CdC_2_ and Zr_3_SbC_2_ phases will accept shear deformation along the *b*- and *c*-axes when adequate stress is applied to the *a*-axis of the crystals.

### 3.2. Theoretical Mechanical Properties of Zr_3_CdC_2_ and Zr_3_SbC_2_ Phases

In order to clearly understand the mechanical properties of the Zr_3_CdC_2_ and Zr_3_SbC_2_ phases, the bulk modulus (*B*), shear modulus (*G*), Young’s modulus (*E*), Pugh’s ratio, and Vickers hardness (*H*) were calculated based on the equations (*S* is the elastic softness constant) [33,34,35,36,37,38,39,40,41]:

(3) $$B_{V}=(2(C_{11}+2C_{12}+4C_{13}+C_{33})/9$$

(4) $$B_{R}=1/(2S_{11}+2S_{12}+4S_{13}+S_{33})$$

(5) $$G_{V}=(3.5C_{11}-2.5C_{12}-2C_{13}+C_{33}+6C_{44})/15$$

(6) $$G_{R}=15/(8S_{11}-10S_{12}-8S_{13}+4S_{33}+6S_{44})$$

(7) $$B=(B_{V}+B_{R})/2$$

(8) $$G=(G_{V}+G_{R})/2$$

(9) $$H_{V}^{G}=0.1769G-2.899$$

**Figure 3:**
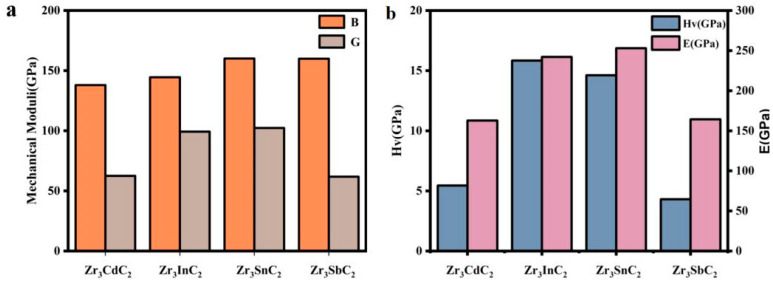
Variation trend of (**a**) bulk moduli (*B*) and shear moduli (*G*), and (**b**) Young’s moduli (E) and Vickers hardnesses (Hv) of Zr_3_AC_2_ (A = Cd, In, Sn, and Sb) phases.

The calculated bulk moduli, shear moduli, and Vickers hardnesses of the Zr_3_CdC_2_ and Zr_3_SbC_2_ phases are listed in Table 3, compared to those of the Zr_3_InC_2_ and Zr_3_SnC_2_ phases. Also, the changing tendencies of the bulk moduli, shear moduli, Young’s moduli, and Vickers hardness of the Zr_3_AC_2_ (A = Cd, In, Sn, and Sb) phases are shown in Figure 3. It is seen that the bulk modulus shows a monotonic increase from 137.942 to 160.068 GPa when the A-site element goes from Cd to In and finally to Sn (Figure 3a). For the Zr_3_SnC_2_ and Zr_3_SbC_2_ phases, they have larger bulk moduli of 160.068 and 159.913 GPa, respectively. Whereas for the shear modulus, the Zr_3_SbC_2_ and Zr_3_CdC_2_ phases have lower values of 61.810 and 62.477 GPa than those of Zr_3_InC_2_ and Zr_3_SnC_2_ of 95.251 and 102.306 GPa, respectively. According to the calculated Pugh’s ratio, Zr_3_CdC_2_ (2.208) and Zr_3_SbC_2_ (2.587) are the ductile phases (B/G > 1.75), while Zr_3_InC_2_ (1.455) and Zr_3_SnC_2_ (1.565) belong to the brittle phases. As a result, the Zr_3_InC_2_ (15.842 GPa) and Zr_3_SnC_2_ (14.625 GPa) phases have higher Vickers hardnesses than those of Zr_3_CdC_2_ (5.488 GPa) and Zr_3_SbC_2_ (4.309 GPa), as shown in Figure 3b.

Additionally, the bond length, bond population, and Bader charge (ΔQ) of the Zr_3_CdC_2_ and Zr_3_SbC_2_ phases were calculated, as listed in Table 4. Here, Zr1 is an atom far away from the atomic layer A, and Zr2 is an atom near the atomic layer A. It can be seen that the comprehensive bond populations of A-site atoms In (0.87 and 1.24) and Sn (0.87 and 1.23) are all larger than those containing Cd atoms (0.85 and 1.24), indicating that the covalent interaction between Zr and In/Sn elements is strong. Because Zr_3_InC_2_ and Zr_3_SnC_2_ crystals have stronger covalent bonds inside, they show a higher elastic modulus and Vickers hardness. Whereas, for the Zr_3_SbC_2_ phase, though its interatomic population number is larger (0.88 and 1.24), its interatomic bond length (2.384 Å and 2.268 Å) is larger than those of Zr_3_InC_2_ (2.383 Å and 2.245 Å) and Zr_3_SnC_2_ (2.380 Å and 2.260 Å). Generally speaking, the longer the bond length, the lower the bond energy and the lower the Vickers hardness. Additionally, the total electron gain/loss of electrons in the crystals of Zr_3_CdC_2_ (1.692 and 2.726) and Zr_3_SbC_2_ (1.700 and 2.658) is greater than those of Zr_3_InC_2_ (1.698 and 2.703) and Zr_3_SnC_2_ (1.577 and 2.526), which indicates that their ionic properties are stronger and their covalent properties are weaker; that is, their Vickers hardness values are lower than those of Zr_3_InC_2_ and Zr_3_SnC_2_. The relatively high atomic charge transfer indicates the main ionic bond characteristics. The number of charges transferred by Zr1 is higher than that transferred by Zr2, which indicates that its covalence is weak. Interestingly, this conclusion is self-consistent with the long bond length and weak bond energy of Zr1-C [42].

### 3.3. Synthesis and Microstructure Characterization of Zr_3_CdC_2_ and Zr_3_SbC_2_ Phases

**Figure 4:**
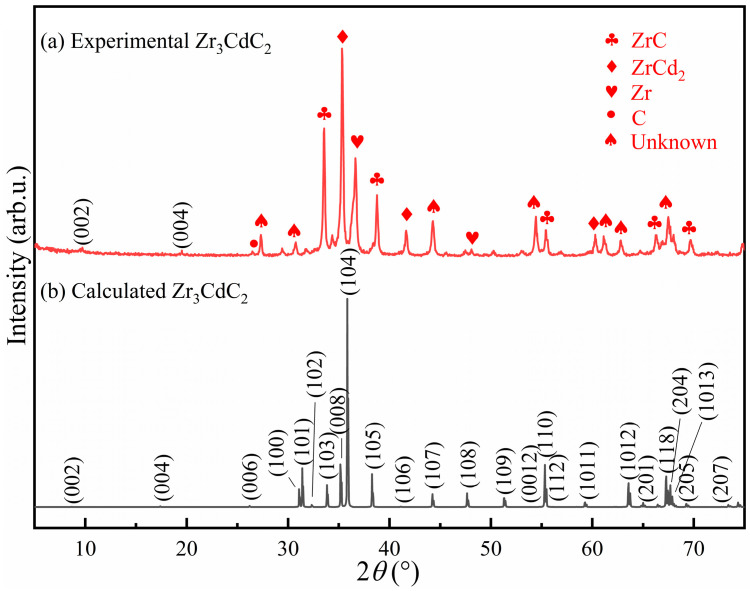
(**a**) Experimental and (**b**) theoretically calculated X-ray diffraction (XRD) patterns of Zr_3_CdC_2_ samples. The sample was synthesized at 850 °C with the molar ratio of Zr:Cd:C = 3:1.5:1.5.

**Figure 5:**
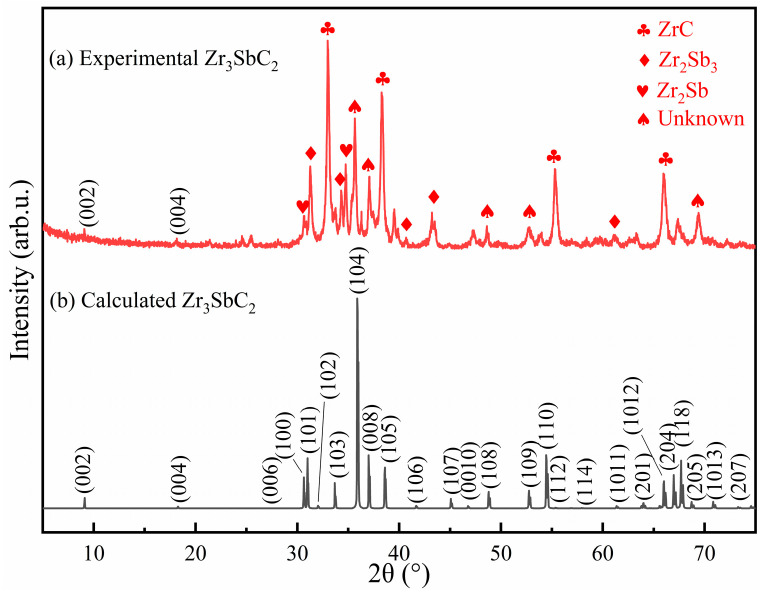
(**a**) Experimental and (**b**) theoretically calculated XRD patterns of Zr_3_SbC_2_ samples. The sample was synthesized at 1300 °C with the molar ratio of Zr:Sb:C = 3:1.5:1.5.

Initially, in an attempt to synthesize the Zr_3_CdC_2_ and Zr_3_SbC_2_ phases by spark plasma sintering, mixed powders with the molar ratio of Zr:Cd/Sb:C = 3:1.5:1.5 were sintered based on the consideration of easy evaporation of Cd/Sb and C deficiency in the *P6_3_/mmc* hexagonal crystals. Considering the synthesis temperature of Ti_2_CdC (750 °C [22]), Zr_3_InC_2_ (1400 °C [21]) and Zr_3_SnC_2_ (1200 °C [19]), the sintering temperatures of complexes were initially designed as 850 °C and 1300 °C, respectively. The examined X-ray diffraction (XRD) patterns acquired from the synthesized samples are displayed in Figure 4a and Figure 5a. In Figure 4a, in combination with the PDF cards, it is determined that a large amount of Zr, ZrC, ZrCd_2_, and unknown phases exist in the cadmium-containing sample; while, in Figure 5a, it is judged that ZrC, Zr_2_Sb_3_, Zr_2_Sb, and unknown phases are in the antimony-containing sample. Significantly, in comparison with the theoretical XRD patterns, as shown in Figure 4b and Figure 5b, the (002) and (004) diffraction peak positions of Zr_3_CdC_2_ and Zr_3_SbC_2_ have a similar degree of rightward shift. This might be due to the occurrence of crystal defects in the Zr_3_CdC_2_ and Zr_3_SbC_2_ phases, resulting in smaller lattice parameters. Anyhow, the (002) and (004) diffraction planes of Zr_3_CdC_2_ and Zr_3_SbC_2_ can still be presumed to be in greater accord with the theoretical results. Therefore, it is expected that the Zr_3_CdC_2_ and Zr_3_SbC_2_ phases have been synthesized. Furthermore, in order to enhance the purity of the samples, the mixture powders were sintered at different temperatures, and the different molar ratios of the mixture powders were designed and annealed at the optimal temperature for comparison. Figure S1 shows the XRD patterns of the cadmium-containing samples synthesized at 650–1050 °C with the molar ratio of Zr:Cd:C = 3:1.5:1.5. And Figure S2 shows the XRD patterns of the antimony-containing samples synthesized at 900–1400 °C with the molar ratio of Zr:Sb:C = 3:1.5:1.5. By comparing the XRD patterns in Figure S1, it can be found that only at 850 °C does the cadmium-containing sample have the least impurity phases, and the XRD pattern is closest to the theoretical result in Figure 4b. Therefore, 850 °C is considered to be the optimum sintering temperature for the Zr_3_CdC_2_ phase. Similarly, the optimum sintering temperature for synthesizing the Zr_3_SbC_2_ phase was determined as 1300 °C. Continuously, for the Zr_3_CdC_2_ phase, the mixed powders with the molar ratios of Zr:Cd:C = 3:(1.4–1.8):2 were sintered at 850 °C, and the examined XRD patterns are shown in Figure S3. It is seen that no obvious existence of the Zr_3_CdC_2_ phase could be determined. Then, the molar ratios of the mixture powders were modified to be 3:1.5:(1.5–2.25), and the samples were sintered at the optimum temperature of 850 °C. It is seen that there is no remarkable enhancement of purity of the Zr_3_CdC_2_ phase, as shown in Figure S4. It seems that the optimal molar ratio is 3:1.5:1.5 to synthesize the Zr_3_CdC_2_ phase. Similarly, for the Zr_3_SbC_2_ phase, the mixed powders with the molar ratios of Zr:Sb:C = 3:(1.5–1.95):2 were sintered at 1300 °C. By comparing the XRD patterns of samples sintered with the different molar ratios, the purity of the Zr_3_SbC_2_ phase was not obviously enhanced, as shown in Figure S5. Therefore, the optimized molar ratio of synthesizing the Zr_3_SbC_2_ phase is confirmed as Zr:Sb:C = 3:1.5:1.5. Owing to the existence of too many impurities in the sintered samples, it is difficult to refine the obtained XRD patterns by using the Rietveld method. Anyhow, in order to richen the information of the new phases of Zr_3_CdC_2_ and Zr_3_SbC_2_, the theoretical crystal constants, atomic positions, and the XRD data are given and listed in Tables S1–S3.

**Figure 6:**
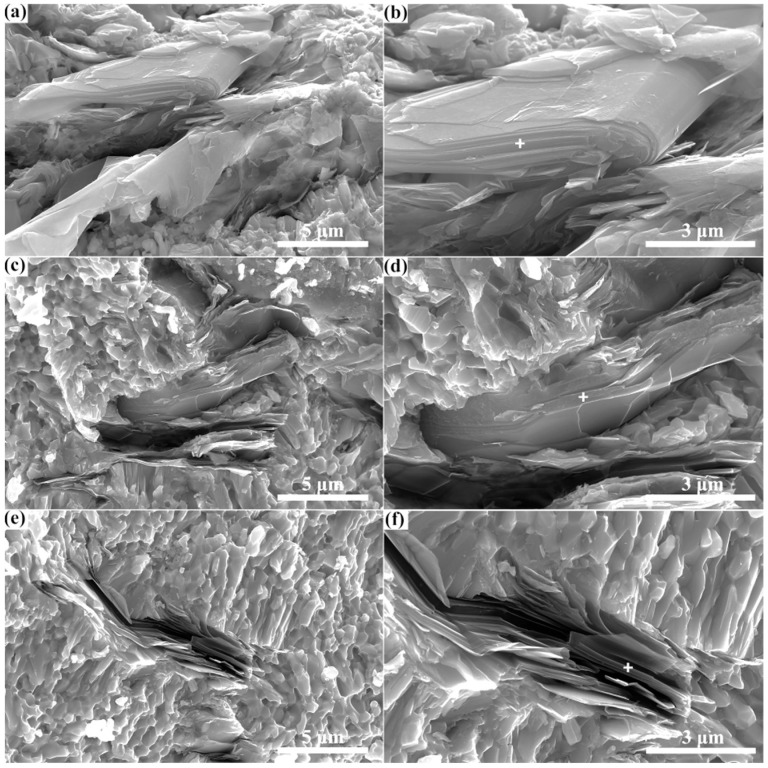
(**a**–**f**) Scanning electron microscopy (SEM) images of fracture surface of the Zr_3_CdC_2_ sample. The testing positions of energy dispersive spectroscopy (EDS) analysis are labeled by white cross in images (**b**,**d**,**f**).

**Figure 7:**
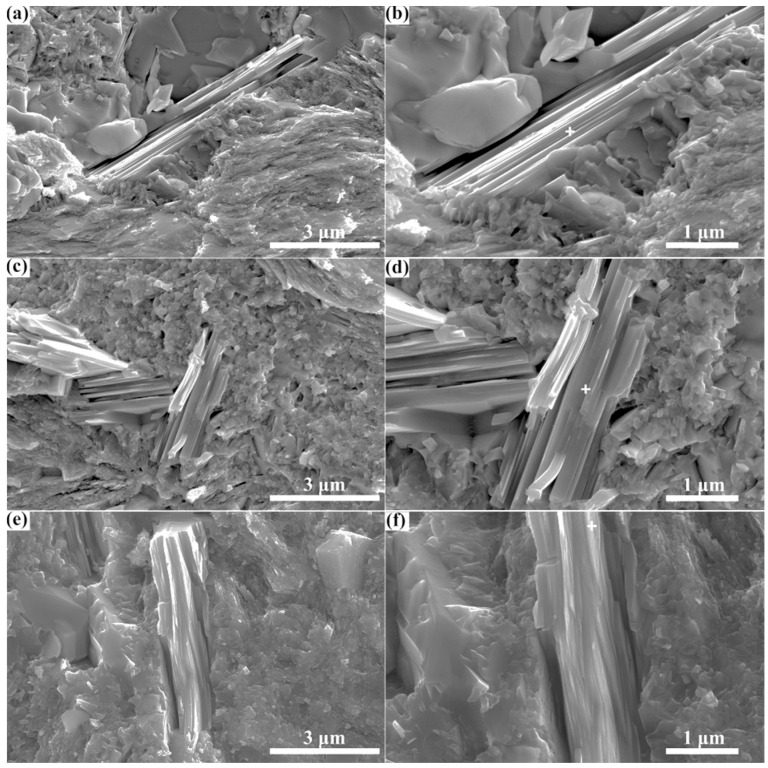
(**a**–**f**) SEM images of fracture surface of Zr_3_SbC_2_ sample. The testing positions of EDS analysis are labeled by the white cross in images (**b**,**d**,**f**).

In addition, in order to characterize the Zr_3_CdC_2_ and Zr_3_SbC_2_ grains in the sintered samples, the fracture surfaces of sintered bulks that underwent the shear loading were observed by using the scanning electron microscope, as shown in Figure 6 and Figure 7. Clearly, the grains with a typical nanolaminar character of MAX phases in the sintered bulks are the Zr_3_CdC_2_ and Zr_3_SbC_2_ phases (Figure 6a–f and Figure 7a–f), which are the same as other typical MAX phases of Ti_3_AlC_2_ and Ti_3_SiC_2_ [43,44]. Obvious bending and twisting phenomena were observed in these grains. Many equiaxed carbide grains are nearby. Additionally, the testing positions of the energy dispersive spectroscopy (EDS) analysis are labeled by white crosses in Figure 6 and Figure 7b,d,f, and the results are displayed in Table 5 and Table 6. It is confirmed that the average element ratios are Zr:Cd = 3:0.976 for Zr_3_CdC_2_ grains and Zr:Sb = 3:1.012 for Zr_3_SbC_2_ grains, respectively, very close to the ratio of 3:1. Here, no data of the C element was collected, as the EDS analysis could not guarantee the accuracy of the C element.

Therefore, based on the above results, it is believed that the Zr_3_CdC_2_ and Zr_3_SbC_2_ phases could stably exist and be successfully synthesized. Combined with the above theoretical calculations, the pure Zr_3_CdC_2_ and Zr_3_SbC_2_ MAX phases are shown to have excellent machinability. However, due to the limitation of sintering, it is rather difficult to purify the Zr_3_CdC_2_ and Zr_3_SbC_2_ phases, and it is greatly worth it for us to explore new synthesis methods to improve the purity of the two MAX phases.

## 4. Conclusions

Two new ternary laminar MAX phases of Zr_3_CdC_2_ and Zr_3_SbC_2_ were predicted and synthesized. The obtained results are listed as follows:

1. It was confirmed that the Zr_3_CdC_2_ and Zr_3_SbC_2_ phases belonged to the space group of *P*6_3_*/mmc* with the hexagonal crystal structure. The calculated crystal parameters of Zr_3_CdC_2_ were *a* = 3.319 Å and *c* = 20.393 Å, and those of Zr_3_SbC_2_ were *a* = 3.367 Å and *c* = 19.413 Å. The calculated formation energies of Zr_3_CdC_2_ and Zr_3_SbC_2_ were −1.104 and −1.306 eV/atom, respectively, which confirm the thermal stability of the two phases. The absence of imaginary frequencies in the acoustic branches of the phonon band structure and the calculation results using the Born–Huang criterion confirmed the thermodynamic and mechanical stability of the two MAX phases. Additionally, the calculated Young’s moduli of Zr_3_CdC_2_ and Zr_3_SbC_2_ were 162.846 GPa and 164.265 GPa, respectively. The theoretical Vickers hardnesses of the Zr_3_CdC_2_ (5.448 GPa) and Zr_3_SbC_2_ (4.309 GPa) phases were low due to the weaker covalent bonds, exhibiting excellent potential machinability.
2. Through spark plasma sintering, composites containing the Zr_3_CdC_2_ and Zr_3_SbC_2_ phases could be synthesized at temperatures of 850 °C and 1300 °C, respectively. The optimal molar ratio of Zr:Cd/Sb:C was determined as 3:1.5:1.5. Based on the SEM micrographs, the nanolayered characters of the Zr_3_CdC_2_ and Zr_3_SbC_2_ grains were determined. The average element ratios were Zr:Cd = 3:0.976 for Zr_3_CdC_2_ grains and Zr:Sb = 3:1.012 for Zr_3_SbC_2_ grains, respectively, very close to the ratio of 3:1.

## Figures and Tables

**Table 1:** Calculated lattice parameters (Å) and formation energies (eV/atom) of Zr_3_AC_2_ (A = Cd, In, Sn, and Sb) phases.

Phase	*a* (Å)	*c* (Å)	Δ*H* (eV/atom)
Zr_3_CdC_2_	3.319	20.393	−1.104
Zr_3_InC_2_	3.337 3.352 [21]	20.251 20.252 [21]	−1.220
Zr_3_SnC_2_	3.348 3.359 [19]	19.870 19.876 [19]	−1.282
Zr_3_SbC_2_	3.367	19.413	−1.306

**Table 2:** Calculated elastic constants of C*_ij_* of Zr_3_AC_2_ (A = Cd, In, Sn, and Sb) phases.

Phase	C_11_ (GPa)	C_12_ (GPa)	C_33_ (GPa)	C_13_ (GPa)	C_44_ (GPa)
Zr_3_CdC_2_	283.717	94.155	228.795	67.108	33.936
Zr_3_InC_2_	310.372	81.969	248.679	68.846	84.965
Zr_3_SnC_2_	286.005	109.654	290.242	89.750	118.166
Zr_3_SbC_2_	239.549	108.369	252.822	126.183	62.190

**Table 3:** Calculated mechanical moduli of *B*, *E*, *G*, Pugh’s ratio of *B*/*G*, Vickers hardness of *H_V_*, melting point of T_m_, and Debye temperatures of T_D_ of Zr_3_AC_2_ (A = Cd, In, Sn, and Sb) phases.

Phase	*B* (GPa)	*G* (GPa)	*E* (GPa)	*B*/*G*	*H_V_* (GPa)	T_m_ (K)	T_D_ (K)
Zr_3_CdC_2_	137.942	62.477	162.846	2.208	5.448	1548.344	392.7
Zr_3_InC_2_	144.437	95.251	242.262	1.455	15.842	1658.135	488.9
Zr_3_SnC_2_	160.068	102.306	253.013	1.565	14.625	1647.378	493.7
Zr_3_SbC_2_	159.913	61.810	164.265	2.587	4.309	1451.880	386.2

**Table 4:** The calculated bond length, bond population, and Bader charge (ΔQ) of Zr_3_AC_2_ (A = Cd, In, Sn, and Sb) phases.

Phase	Zr1-C Length (Å)	Zr1-C Population	Zr2-C Length (Å)	Zr2-C Population	ΔQ_Zr1_	ΔQ_Zr2_	ΔQC
Zr3CdC2	2.377	0.85	2.242	1.24	1.591	1.135	−1.692
Zr3InC2	2.383	0.87	2.245	1.24	1.592	1.111	−1.698
Zr3SnC2	2.380	0.87	2.260	1.23	1.578	0.948	−1.577
Zr3SbC2	2.384	0.88	2.268	1.24	1.642	1.016	−1.700

**Table 5:** EDS analysis results of Zr_3_CdC_2_ grains at the test positions in Figure 6b,d,f.

Element	At.% in Figure 6b	At.% in Figure 6d	At.% in Figure 6f
Zr	75.67	75.31	75.38
Cd	24.33	24.69	24.62

**Table 6:** EDS analysis results of Zr_3_SbC_2_ grains at the test positions in Figure 7b,d,f.

Element	At.% in Figure 7b	At.% in Figure 7d	At.% in Figure 7f
Zr	74.48	74.10	75.74
Sb	25.52	25.90	24.26

## Data Availability

All data that support the findings of this study are available from the corresponding author.

## Supplementary Materials

The following supporting information can be downloaded at: https://www.mdpi.com/article/10.3390/ma17071556/s1. Figure S1: XRD patterns of Zr_3_CdC_2_ samples with the molar ratio of Zr:Cd:C = 3:1.5:1.5 synthesized at the different sintering temperature: (a) 1050 °C, (b) 950 °C, (c) 850 °C, (d) 750 °C, and (e) 650 °C. Figure S2: XRD patterns of Zr_3_SbC_2_ samples with the molar ratio of Zr:Sb:C = 3:1.5:1.5 synthesized at the different sintering temperature: (a) 1400 °C, (b) 1300 °C, (c) 1200 °C, (d) 1100 °C, (e) 1000 °C, and (f) 900 °C. Figure S3: XRD patterns of Zr_3_CdC_2_ samples synthesized at 850 °C with the different molar ratio of Zr, Cd, and C: (a) 3:1.8:2, (b) 3:1.7:2, (c) 3:1.6:2, (d) 3:1.5:2, and (e) 3:1.4:2. Figure S4: XRD patterns of Zr_3_CdC_2_ samples synthesized at 850 °C with the different molar ratio of Zr, Cd, and C: (a) 3:1.5:2.25, (b) 3:1.5:2, (c) 3:1.5:1.75, and (d) 3:1.5:1.5. Figure S5: XRD patterns of Zr_3_SbC_2_ samples synthesized at 1300 °C with the different molar ratio of Zr, Sb, and C: (a) 3:1.95:1.5, (b) 3:1.8:1.5, (c) 3:1.6:1.5, and (d) 3:1.5:1.5. Table S1: Theoretical crystal parameters and atomic positons of Zr_3_CdC_2_ and Zr_3_SbC_2_ phases. Table S2: Calculated data of reflections, 2*θ*, *d*-spacing, and intensities of Zr_3_CdC_2_ phase. Table S3: Calculated data of reflections, 2*θ*, *d*-spacing, and intensities of Zr_3_SbC_2_ phase.
