# Supplementary material for: Theoretical Prediction and Experimental Synthesis of Zr3AC2 (A = Cd, Sb) Phases
Source: Materials (Basel). 2024 Mar 28;17(7):1556. doi: 10.3390/ma17071556 (PMC11012513; doi:10.3390/ma17071556)
Supplement: Supplementary file 1 [file materials-17-01556-s001.zip › materials-2912219-supplementary.pdf]

Supplementary Information

Jia Luo[1,†], Fengjuan Zhang[1,†], Bo Wen[1,†], Qiqiang Zhang[1], Longsheng Chu[1], Yanchun Zhou[2,*], Qingguo Feng[1,*], Chunfeng Hu[1,*]

[1]*Key Laboratory of Advanced Technologies of Materials, Ministry of Education, School of Materials Science and Engineering, Southwest Jiaotong University, Chengdu 610031, China*

[2]*School of Materials Science and Engineering, Zhengzhou University, Zhengzhou 450001, China*

†The authors contribute equally to the work
*Corresponding authors:
Dr. Yanchun Zhou
School of Materials Science and Engineering
Zhengzhou University
Zhengzhou 450001, China
E-mail address: yczhou@alum.imr.ac.cn

Dr. Qingguo Feng
School of Materials Science and Engineering
Southwest Jiaotong University
Chengdu 610031, China
E-mail address: qfeng@swjtu.edu.cn

Dr. Chunfeng Hu
School of Materials Science and Engineering
Southwest Jiaotong University
Chengdu 610031, China
E-mail address: chfhu@live.cn

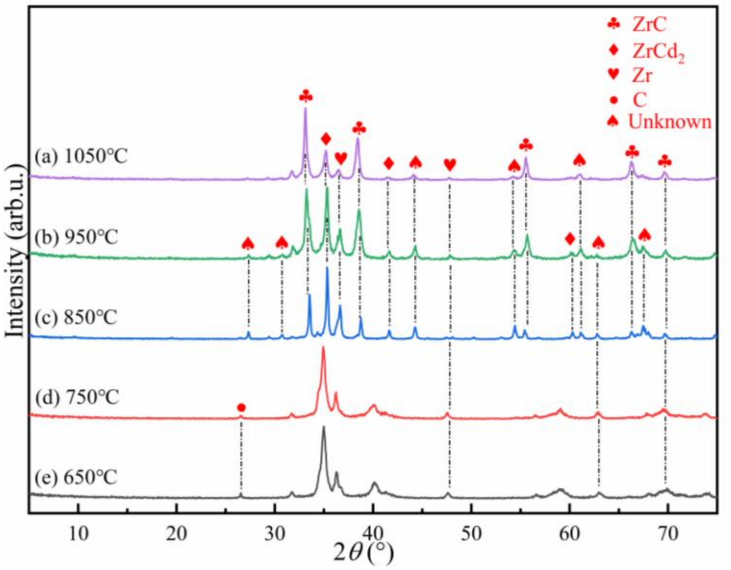

Figure S1. XRD patterns of Zr_3CdC_2 samples with the molar ratio of Zr : Cd : C = 3 : 1.5 : 1.5 synthesized at the different sintering temperature: (a) 1050 ℃, (b) 950 ℃, (c) 850 ℃, (d) 750 ℃, and (e) 650 ℃.

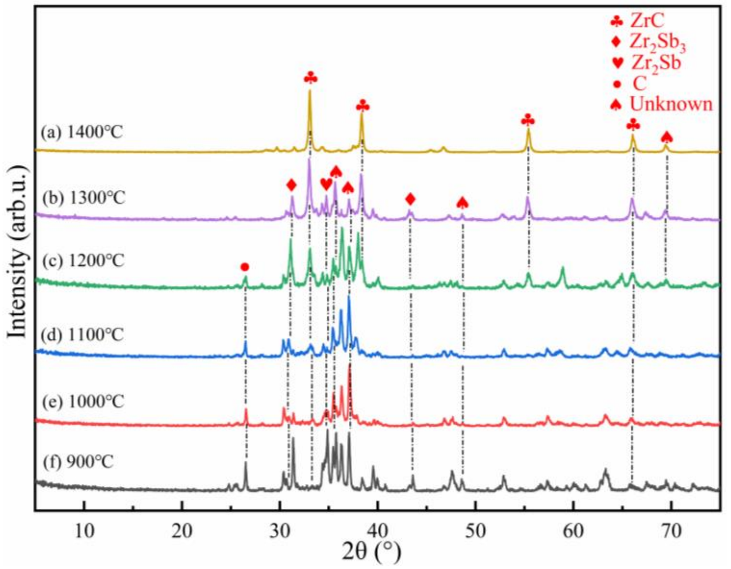

Figure S2. XRD patterns of Zr_3SbC_2 samples with the molar ratio of Zr : Sb : C = 3 : 1.5 : 1.5 synthesized at the different sintering temperature: (a) 1400 ℃, (b) 1300 ℃, (c) 1200 ℃, (d) 1100 ℃, (e) 1000 ℃, and (f) 900 ℃.

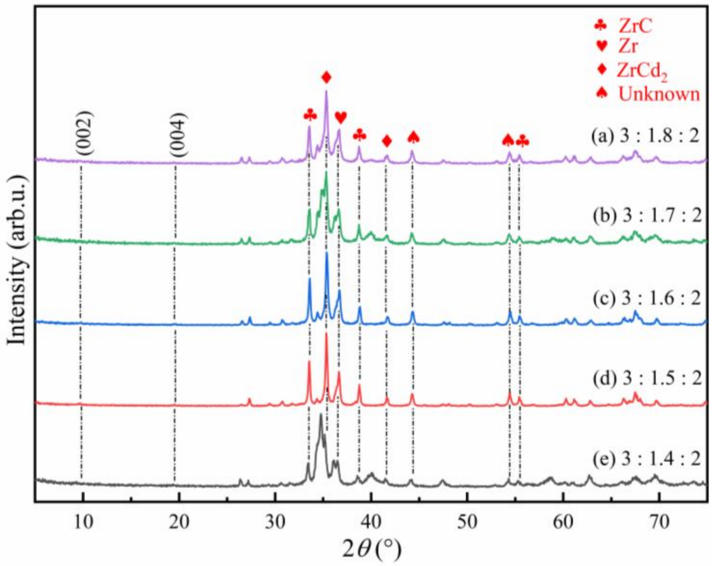

Figure S3. XRD patterns of Zr_3CdC_2 samples synthesized at 850 °C with the different molar ratio of Zr, Cd, and C: (a) 3 : 1.8 : 2, (b) 3 : 1.7 : 2, (c) 3 : 1.6 : 2, (d) 3 : 1.5 : 2, and (e) 3 : 1.4 : 2.

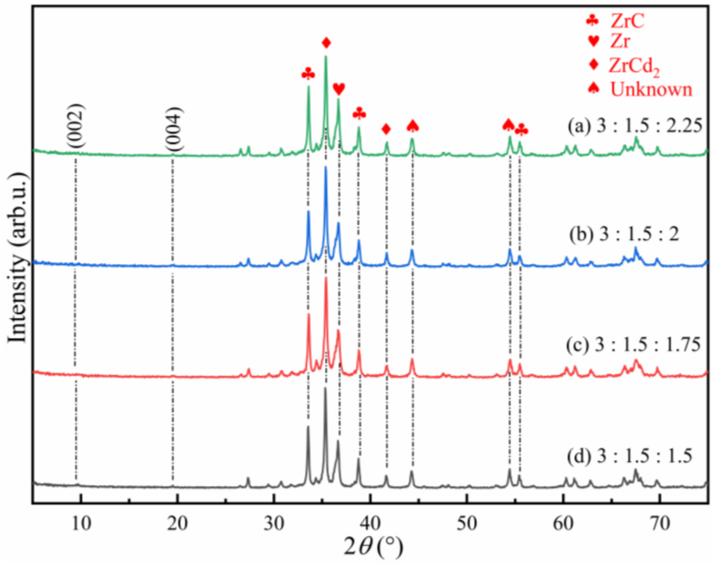

Figure S4. XRD patterns of Zr_3CdC_2 samples synthesized at 850 °C with the different molar ratio of Zr, Cd, and C: (a) 3 : 1.5 : 2.25, (b) 3 : 1.5 : 2, (c) 3 : 1.5 : 1.75, and (d) 3 : 1.5 : 1.5.

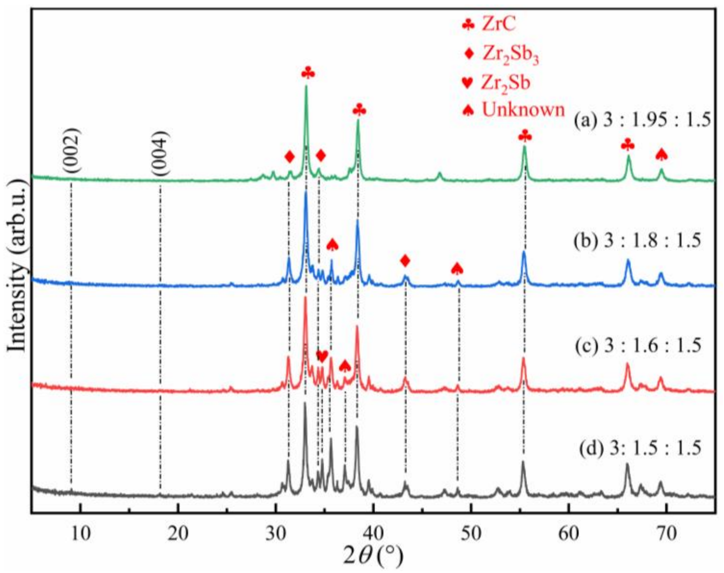

Figure S5. XRD patterns of Zr_3SbC_2 samples synthesized at 1300 °C with the different molar ratio of Zr, Sb, and C: (a) 3 : 1.95 : 1.5, (b) 3 : 1.8 : 1.5, (c) 3 : 1.6 : 1.5, and (d) 3 : 1.5 : 1.5.

Table S1. Theoretical crystal parameters and atomic positons of Zr_3CdC_2 and Zr_3SbC_2 phases.

Chemical formula	Zr_3CdC_2		Zr_3SbC_2
M (g/mol)	410.104		419.453
Crystal system		Hexagonal	
Space group		*P*6$_3$*/mmc* (No.194)	
a (Å)	3.319		3.367
c (Å)	20.393		19.413
V (Å^3)	194.491		190.603
Z		2	
Atomic positions	x	y	z
M1	0	0	0
M2	1/3	2/3	0.126125[Cd], 0.131194[Sb]
A	0	0	1/4
C	1/3	2/3	0.568994[Cd], 0.571044[Sb]

Table S2. Calculated data of reflections, 2θ, d-spacing, and intensities of Zr_3CdC_2 phase.

(*hkl*)	$2\theta_{Cal.}$ (°)	$d_{Cal.}$ (Å)	$I/I_{0Cal.}$ (%)
(0 0 2)	8.665	10.197	0.104
(0 0 4)	17.380	5.098	0.338
(0 0 6)	26.198	3.399	0.514
(1 0 0)	31.089	2.874	8.698
(1 0 1)	31.404	2.846	17.640
(1 0 2)	32.333	2.767	1.165
(1 0 3)	33.831	2.647	11.093
(0 0 8)	35.177	2.549	26.691
(1 0 4)	35.834	2.504	100.000
(1 0 5)	38.276	2.350	15.915
(1 0 6)	41.093	2.195	0.230
(1 0 7)	44.230	2.046	7.233
(0 0 10)	44.385	2.039	0.174
(1 0 8)	47.643	1.907	7.326
(1 0 9)	51.300	1.779	6.645
(0 0 12)	53.906	1.699	0.049
(1 0 10)	55.178	1.663	0.039
(1 1 0)	55.312	1.660	25.671
(1 1 2)	56.104	1.638	0.046
(1 1 4)	58.436	1.578	0.085
(1 0 11)	59.263	1.558	3.497
(1 1 6)	62.200	1.491	0.150
(1 0 12)	63.546	1.463	17.360
(0 0 14)	63.850	1.457	0.006
(2 0 0)	64.820	1.437	1.038
(2 0 1)	65.000	1.434	2.204
(2 0 2)	65.540	1.423	0.105
(2 0 3)	66.435	1.406	1.679
(1 1 8)	67.264	1.391	23.813
(2 0 4)	67.678	1.383	14.852
(1 0 13)	68.029	1.377	2.169
(2 0 5)	69.260	1.355	2.565
(2 0 6)	71.171	1.324	0.041
(1 0 14)	72.717	1.299	0.177
(2 0 7)	73.402	1.289	1.565
(1 1 10)	73.515	1.287	0.191
(0 0 16)	74.364	1.275	3.395

Table S3. Calculated data of reflections, 2θ, d-spacing, and intensities of Zr_3SbC_2 phase.

(hkl)	$2\theta_{Cal.}$ (°)	$d_{Cal.}$ (Å)	$I/I_{0Cal.}$ (%)
(0 0 2)	9.103	9.707	2.584
(0 0 4)	18.265	4.853	0.589
(0 0 6)	27.546	3.236	0.015
(1 0 0)	30.635	2.916	9.770
(1 0 1)	30.987	2.884	18.829
(1 0 2)	32.023	2.793	1.142
(1 0 3)	33.685	2.658	8.302
(1 0 4)	35.899	2.499	100.000
(0 0 8)	37.015	2.427	22.327
(1 0 5)	38.582	2.332	18.753
(1 0 6)	41.662	2.166	1.173
(1 0 7)	45.078	2.010	3.457
(0 0 10)	46.755	1.941	0.883
(1 0 8)	48.783	1.865	7.825
(1 0 9)	52.744	1.734	8.518
(1 1 0)	54.458	1.684	27.110
(1 1 2)	55.340	1.659	0.310
(0 0 12)	56.868	1.618	0.156
(1 0 10)	56.937	1.616	0.0205
(1 1 4)	57.932	1.591	0.192
(1 0 11)	61.352	1.510	1.061
(1 1 6)	62.099	1.493	-
(2 0 0)	63.785	1.458	1.232
(2 0 1)	63.986	1.454	2.382
(2 0 2)	64.587	1.442	0.129
(2 0 3)	65.582	1.422	1.310
(1 0 12)	65.983	1.415	14.784
(2 0 4)	66.961	1.396	15.661
(0 0 14)	67.490	1.387	0.188
(1 1 8)	67.680	1.383	22.737
(2 0 5)	68.715	1.365	3.191
(2 0 6)	70.830	1.329	0.259
(1 0 13)	70.835	1.329	3.087
(2 0 7)	73.295	1.290	0.798
(1 1 10)	74.549	1.272	1.277
